# Methodological Quality of Manuscripts Reporting on the Usability of Mobile Applications for Pain Assessment and Management: A Systematic Review

**DOI:** 10.3390/ijerph17030785

**Published:** 2020-01-27

**Authors:** Ana F. Almeida, Nelson P. Rocha, Anabela G. Silva

**Affiliations:** 1 Institute of Electronics and Informatics Engineering of Aveiro (IEETA), Campus Universitário de Santiago, 3810-193 Aveiro, Portugalnpr@ua.pt (N.P.R.); 2 Department of Medical Sciences, Universidade de Aveiro - Edifício 30, Agras do Crasto - Campus Universitário de Santiago, 3810-193 Aveiro, Portugal; 3 School of Health Sciences, Universidade de Aveiro - Edifício 30, Agras do Crasto - Campus Universitário de Santiago, 3810-193 Aveiro, Portugal; 4 Center for Health Technology and Services Research, Universidade de Aveiro (CINTESIS.UA), Campus Universitário de Santiago, 3810-193 Aveiro, Portugal

**Keywords:** pain, app, mobile app, mobile application, application, usability

## Abstract

Background: There has been increasing use of mobile mHealth applications, including pain assessment and pain self-management apps. The usability of mHealth applications has vital importance as it affects the quality of apps. Thus, usability assessment with methodological rigor is essential to minimize errors and undesirable consequences, as well as to increase user acceptance. Objective: this study aimed to synthesize and evaluate existing studies on the assessment of the usability of pain-related apps using a newly developed scale. Methods: an electronic search was conducted in several databases, combining relevant keywords. Then titles and abstracts were screened against inclusion and exclusion criteria. The eligible studies were retrieved and independently screened for inclusion by two authors. Disagreements were resolved by discussion until consensus was reached. Results: a total of 31 articles were eligible for inclusion. Quality assessment revealed that most manuscripts did not assess usability using valid instruments or triangulation of methods of usability assessment. Most manuscripts also failed to assess the three domains of usability (effectiveness, efficiency and satisfaction). Conclusions: future studies should consider existing guidelines on usability assessment design, development and assessment of pain-related apps.

## 1. Introduction

Pain is a global health problem, affecting all populations, regardless of age, sex, income, race/ethnicity, or geography [1]. It represents one of the main motives for seeking healthcare and a huge clinical, social and economic problem [1], conditioning life activities and being responsible for morbidity, absence from work, and temporary or permanent disability. Inadequate pain assessment and poor management have an impact on patients’ psychological status, often resulting in anxiety and depression, and contribute to the longstanding maintenance of pain [2]. Therefore, choosing an appropriate instrument to assess pain is very important [2], and constitutes the first step for effective pain management [3].

Over the years, there has been a global push towards using information technologies to address health needs [4], particularly as the mobile Health (mHealth) application (app) market grows. A study of the status and trends of the mHealth market reported that in 2016 there were about 259.000 mHealth apps available on major app stores [5]. Furthermore, there is an increasing number of smartphone apps devoted to pain assessment and pain self-management strategies available in app stores [6,7,8]. The first three pain-related apps that appear in the Play Store when searched using the word “pain”(search performed on the 15th of January 2020 on a smartphone) were Manage my Pain, Pain Diary and Pain Companion and the description of these apps refers that they have been downloaded 100,000, 50,000, 10,000 or more times, respectively, suggesting that a considerable number of persons is using them. Recent reviews have evaluated the quality of pain-related apps [9,10], focusing on its feasibility, acceptability or content and functioning. However, a major aspect to consider when assessing the quality of an app is usability [11], but usability has received less attention. 

The International Organization for Standardization (ISO) 9241-11 [12] defines usability as the “extent to which a system, product or service can be used by specific users to achieve specific goals with effectiveness, efficiency, and satisfaction in a specific context of use”. Effectiveness refers to the accuracy and completeness with which users achieve specified goals, and can be characterized using measures of accuracy and completeness such as task success. Efficiency refers to the resources used concerning the results achieved, and the time needed to complete tasks can be used as an indicator. Satisfaction is the extent to which the user’s physical, cognitive and emotional responses that result from the use of a system, product or service meet the user’s needs and expectations and can be assessed through interviews, focus groups or scales and questionnaires [12]. The assessment of usability can be formative if its main aim is to detect and solve problems or summative if its main aim is to meet the metrics associated with the system, product, or service task and goals [13]. A formative assessment is usually more common at earlier phases of a system or product development, and the type of instruments and procedures used depends on whether the assessment is formative or summative and on the development phase of the system, product, or service [14]. Independently of the phase of development, it is usually good practice to use a combination of approaches for usability assessment, involving, for example, the combination of quantitative or qualitative approaches, health professionals and patients or assessments in the laboratory and real context [15].

Usability assessment with methodological rigor is essential to minimize the likelihood of errors and undesirable consequences, to increase user acceptance, and to develop highly usable systems and products more likely to be successful in the market [16]. The adoption of usable mHealth systems or products will lead to safer and higher quality care, and a higher return of investment for institutions [17]. However, the assessment of usability is a complex process that requires, as referred, a combination of methods for a deeper and comprehensive evaluation of a product/service [18]. The methodological robustness and quality of these methods can have an impact on the results of the usability assessment and, therefore, should be carefully considered when interpreting the results of usability studies. Reviews on mobile applications for pain assessment or management have focused on the quality and content of the mobile applications [19,20] rather than on the quality of the process of assessment of usability. A robust existing scale, the Mobile App Rating Scale, also aims to assess the quality of the mobile apps [21]. A recent systematic review attempted to assess the quality of usability studies using a four-point scale [22] but failed to report on the process of development of the scale, or its reliability or validity, fundamental characteristics of any assessment or measurement instrument. Recently, a 15-items scale was developed to assess the methodological quality of usability studies for eHealth applications or general applications [23]. This scale was developed using a robust process, including a three-round Delphi method with 25 usability experts to generate a first version of the scale, which was then tested for comprehensibility by another three experts to generate a final version of the scale. This final version was shown to be feasible and both valid and reliable [23]. The present review aims to synthesize and evaluate existing studies on the assessment of the usability of pain-related apps using this newly developed scale [23]. 

## 2. Methods 

### 2.1. Study Design

This systematic review followed the Preferred Reporting Items for Systematic Reviews and Meta-Analyses (PRISMA).

#### 2.1.1. Search Strategy and Selection of The Studies

An electronic search was conducted in Academic Search Complete, Scopus, PubMed, ScienceDirect, Web of Science, Scielo and Institute of Electrical and Electronics Engineers (IEEE) Xplore Digital Library on the 10th of May 2019, from database inception to the day the search was conducted. The following combination of words was used for all databases: pain AND (app OR “mobile app” OR “mobile application” OR application) AND usability. To be included in this systematic review studies had to: include the assessment of the usability of a pain-related mobile application as a study aim in any study sample and setting, be a full article published in a peer-reviewed journal/conference proceeding and be written in English, Portuguese, Spanish or French. The pain-related mobile application could target pain assessment, pain intervention or both. For this review, a mobile app was defined as “a software/set or program that runs on a mobile device and performs certain tasks for the user” [24]. Review articles were excluded. 

Two researchers (AGS and AFA) independently reviewed the retrieved references against inclusion criteria. Disagreements were resolved by discussion until consensus was reached.

#### 2.1.2. Data Extraction

All retrieved references were imported into the reference software Mendeley (Elsevier, North Holland) and checked for duplicates. Then titles and abstracts were screened against inclusion and exclusion criteria. Posteriorly, full texts of potentially eligible studies were retrieved and independently screened for inclusion by two authors of this review (AGS and AFA). The agreement was measured using a Cohen’s K. Values below 0.20 indicate no concordance, between 0.21 and 0.39 minimal concordance, between 0.40 and 0.59 weak concordance, between 0.60 and 0.79 moderate concordance, between 0.80 and 0.90 strong concordance and above 0.90 almost perfect concordance [25].

Data from each included manuscript were extracted by one of the authors (AFA) using a standardized form including 1) manuscript authors, 2) name of the app, 3) app aim (i) pain assessment, i.e., one or two-way communication applications mainly intended to monitor pain characteristics or ii) pain management, i.e., applications designed to provide support/deliver pain-related interventions) [26], 4) individuals involved in the usability and characteristics, 5) use case, 6) domain of usability assessed (efficiency, effectiveness and/or satisfaction), 7) procedures for usability assessment, and 8) usability outcomes. The extracted data was checked by the second author (AGS) and the disagreements between authors at any point in the process were resolved through discussion until consensus was achieved. 

Regarding the usability domains, efficiency refers to the resources used concerning the results achieved; effectiveness refers to the accuracy and completeness with which users achieve specified goals, and satisfaction is the extent to which the user’s physical, cognitive and emotional responses that result from the use of a system, product or service meet the user’s needs and expectations [12]. A study was considered to have assessed efficiency if the time needed to complete tasks was reported; effectiveness when measures of accuracy and completeness regarding specified goals were reported (e.g., task success) and satisfaction was assessed through interviews, focus group and scales/questionnaires (e.g., the System Usability Scale (SUS)) [27].

### 2.2. Methodological Quality Assessment 

The methodological quality of included studies was independently assessed by two reviewers (AGS and AFA) using the Critical Assessment of Usability Studies Scale [23]. This is a recently developed scale, which is both valid and reliable (Intraclass Correlation Coefficient—ICC = 0.81) and scores vary between 0 and 100% [23]. This scale is composed of fifteen questions on the procedures used to assess usability: 1) Did the study use valid measurement instruments of usability (i.e., there is evidence that the instruments used assess usability)? 2) Did the study use reliable measurement instruments of usability (i.e., there is evidence that the instruments used have similar results in repeated measures in similar circumstances)? 3) Was there coherence between the procedures used to assess usability (e.g., instruments, context) and study aims? 4) Did the study use procedures of assessment for usability that were adequate to the development stage of the product/service? 5) Did the study use procedures of assessment for usability adequate to study participants’ characteristics? 6) Did the study employ triangulation of methods for the assessment of usability? 7) Was the type of analysis adequate to the study’s aims and variables measurement scale? 8) Was usability assessed using both potential users and experts? 9) Were participants who assessed the product/service usability representative of the experts’ population and/or of the potential user’s population? 10) Was the investigator that conducted usability assessments adequately trained? 11) Was the investigator that conducted usability assessments external to the process of product/service development? 12) Was the usability assessment conducted in the real context or close to the real context where product/service is going to be used? 13) Was the number of participants used to assess usability adequate (whether potential users or experts)? 14) Were the tasks that serve as the base for the usability assessment representative of the functionalities of the product/service? 15) Was the usability assessment based on continuous and prolonged use of the product/service over time? Items 12 and 15 may be considered as not applicable depending on the phase of product development.

A pilot test of three manuscripts was undertaken and results discussed to clarify potential differences regarding the understanding of the scale items before moving to the assessment of the remaining manuscripts. Disagreements were resolved by discussion until reaching a consensus. The agreement was measured using an ICC (Model 2,1) calculated using SPSS version 24, and an ICC of at least 0.7 was considered acceptable [28]. 

## 3. Results

### 3.1. Study Selection

**Figure 1:**
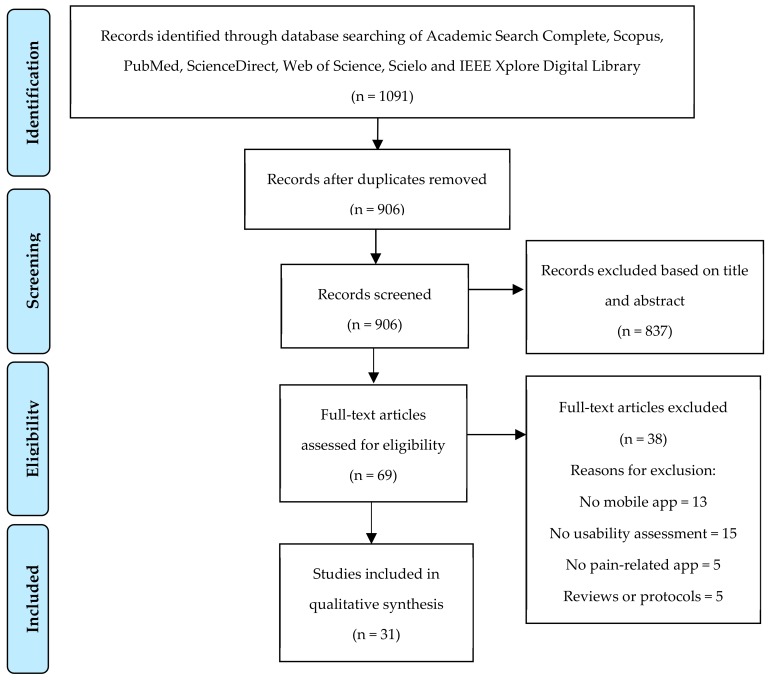
Study selection flowchart (CONSORT).

Searches resulted in 1091 references. After removing duplicates (*n* = 185), 906 references were screened based on title and abstract and 69 full articles were retrieved. Of these, 31 articles were eligible for inclusion (Figure 1). The main reasons for exclusion were: studies without reference to mobile apps (*n* = 13) or no pain-related app (*n* = 5), studies that didn’t assess usability (*n* = 15), studies that were reviews or protocols (*n* = 5). Cohen’s K between the two researchers involved in study selection was 0.77, which indicates moderate concordance. 

### 3.2. Mobile Apps

The 31 manuscripts included covered a total of 32 pain apps (one manuscript assessed three different apps [29]; one manuscript assessed two different apps [19]; two manuscripts assessed the Pain Droid [30,31], and another two assessed the Pain Squad+ [32,33]). Twenty-five of these apps were categorized as pain assessment, and included pain scales and pain diaries to record the users’ pain episodes and pain characteristics. Of these 25 pain assessment apps, 23 were intended for patient use and two for health professionals use (INES-DIO and iPhone pain app). The remaining seven apps were categorized as pain management and included self-management strategies (e.g., meditation, guided relaxation), but all of them included pain assessment features too. 

### 3.3. Usability Assessment 

All manuscripts except three [34,35,36] assessed the domain “satisfaction”. Only eight manuscripts described methods compatible with the assessment of all the three domains of usability (efficiency, effectiveness, and satisfaction), combining objective and subjective indicators, such as interviews, task completion rates and measuring the time participants needed to complete each task [19,29,32,37,38]. However, the results are not clearly and adequately reported in some manuscripts. For example, two studies of De La Vega et al. report, in their methods, recording of errors and use of SUS, however, they do not provide data on the number of errors neither present the final score of the SUS in the results section [39,40]. Similarly, other authors report the use of SUS, but do not provide its final score [31,41,42].

The procedures most commonly used for usability assessment were interviews and open-ended questions, used in 18 manuscripts, as well as verification of completion rates, also used in 18 manuscripts (Table 1). Other approaches to assess usability included using validated questionnaires or scales, observation, task completion times, think aloud and error rate.

Regarding the involvement of end-users in usability assessment, 23 manuscripts assessed apps that were intended for individuals with specific pathologies and used a sample of individuals with characteristics similar to the target group of end-users for usability assessment [19,29,31,32,33,34,36,37,38,40,41,42,43,44,45,46,47,48,49,50,51,52,53]. Participants in these studies included, among others, individuals with fibromyalgia and other rheumatological diseases [37,38,40,43,44], and individuals with headaches [29,32,33,45,46,47,48], cancer-related pain [36,49,50,51], or chronic pain in general [19,41,52,53], individuals with Down syndrome [34] and individuals who are wheelchair users [31,42]. Of these 23 studies, five also targeted specific age groups: four used children or adolescents [32,33,49,51] and one used older individuals [29]. From the remaining manuscripts (*n* = 8), two used children and adolescents [54,55] for usability assessment, four used individuals from the general population [35,39,56,57] and another two used health professionals [58,59]. The overview of the manuscripts analyzed is presented in the Table S1 from the Supplementary file.

**Table 1:** Methods of usability assessment reported in the manuscripts included in this systematic review.

References ^1^	Method of Usability Assessment	*n* (%) (out of 31)
[32,38,39,40,44,48,53,55]	Think aloud approach	8 (25.81%)
[26,29,30,34,36,40,42,45,47,48,49,50,51,52,53,54,55]	Focus group, surveys or interviews	18 (58.06%)
[19,29,31,41,42,43,45,48,55,56,57]	Valid instrument/questionnaire (e.g., System Usability Scale, SUS)	11 (35.48%)
[32,34,37,38,39,40,46,48,53,54]	Observations / recording sessions / field notes	10 (32.26%)
[39,40,55]	Task error rate	3 (9.68%)
[29,32,33,34,35,36,37,39,44,47,49,50,51,54,55,56,57,58]	Completion rates	18 (58.06%)
[19,29,32,34,38,54,55,56]	Recording of task completion times	8 (25.81%)

^1^ The total sum exceeds the number of manuscripts assessed since some used a combination of methods.

### 3.4. Methodological Quality Assessment

The ICC for reviewer’s agreement was 0.71, IC95% [0.42–0.86]. The mean (±SD) score, in percentage, for the 31 manuscripts was 53.93% (SD = 13.01%), ranging between 20% and 73.33%. A more detailed analysis shows that all manuscripts, except one (out of 31) [51] assessed usability using appropriate procedures for the app development phase, but only two (out of 31) reported to have used an investigator adequately trained [35,40], and only one (out of 31) refers that the investigator responsible for usability assessment was external to the product development process [29]. Of the 31 manuscripts included, 11 used valid instruments to assess usability [19,29,31,41,42,43,45,48,55,56,57], but only nine triangulated methods of usability assessment [19,39,40,43,47,49,50,55,57]. The detailed results on the methodological quality assessment are presented in Table 2.

## 4. Discussion

This systematic review found 31 manuscripts assessing the usability of a total of 32 pain-related apps, 25 of which were for pain assessment and seven for pain management. The lower number of mobile apps devoted to pain management may reflect the complexity of pain management, which requires multicomponent interventions but may also suggest that this field requires further development. This is the first systematic review that assesses the methodological quality of studies on the usability assessment of pain-related apps using a scale that is specific for the methodological quality of usability studies and that has been tested for its reliability and validity [23], and results suggest that several important methodological aspects regarding the assessment of usability are not being considered when developing pain-related apps. The complex nature of usability assessment is reflected in the low methodological quality of many studies with 12 (39%) out of 31 manuscripts scoring less than 50% in the methodological quality assessment. With this work, we aim to highlight the need for good practices in the assessment of usability more in line with existing recommendations [12,27]. 

Many of the studies included in the present systematic review fail to use valid (*n* = 18 out of 31) and reliable (*n* = 19 out of 31) measurement instruments. However, validity and reliability are fundamental characteristics of any measurement instrument and an indicator of their quality [60]. Similarly, studies fail to use triangulation of methods (*n* = 22 out of 31), despite the claims that a sound methodology of usability assessment requires the use of combined approaches [18]. Further, when using qualitative approaches for data collection, scarce details were provided in terms of the researchers involved and the procedures used for data collection and data analysis. We strongly suggest that authors follow the existing criteria for reporting qualitative research [61]. A few studies (*n* = 6) do not provide the total score for the instrument used, namely fail to provide the total score for the SUS or state in the methods to have assessed the number of errors, but do not provide this indicator in the results. We highlight the need for systematic planning and report of usability assessment procedures. The full description of all procedures employed for usability assessment may be included as an appendix section if the word limit of some journals prevents the authors from comprehensively reporting procedures and results.

Interestingly, only one manuscript reported having used older adults to assess the usability of a mobile app. Considering that pain prevalence tends to increase with age, mobile apps can have the potential to help health professionals reach a higher number of older adults with pain at lower costs. However, the specificities of this group, including a high number of painful body sites, increased comorbidities, lower digital literacy when compared to younger groups, and potential cognitive deficits in subgroups of older adults, suggest that the design and content of mobile apps need to be specifically designed and tested for this age group.

Other important methodological aspects that most manuscripts did not report was on the experience or training of the researcher involved in the assessment of usability (*n* = 29 out of 31) and whether this person was external to the team developing the app (*n* = 30 out of 31). Nevertheless, most studies employed procedures of usability assessment, such as think-aloud (*n* = 8 out of 31) and focus groups and interviews (*n* = 18 out of 31), that greatly depend on the ability of the researcher to conduct the assessment. Furthermore, if the researcher has a vested interest in the application, this can unintentionally bias the results towards a more favorable outcome. This has been shown for intervention studies, where beliefs and expectations have been found to bias the results towards a higher probability of a type I error (i.e., false-positive result) [62]. The lack of methodological detail on the reports of studies on usability has already been highlighted [23,63]. The exponential growth and the enormous potential of mobile apps to change the paradigm of health interventions, by increasing the access of individuals to health services at lower costs, requires a rigorous and methodologically sound assessment. 

In terms of the usability domains assessed, all but three manuscripts assessed “satisfaction” [34,35,36]. Besides, most manuscripts did not report on the measurement of effectiveness, efficiency, and satisfaction, because they fail to use a combination of methods that allows assessing these three domains [27]. Only eight out of the 31 manuscripts included [19,29,32,37,38,54,55,56] assessed all the three domains of usability, despite existing recommendations to include measures of efficiency, effectiveness, and user satisfaction since a narrower selection of usability measures may lead to unreliable conclusions about the overall usability of the app [64]. Furthermore, there was an inconsistency between what was reported in the methodology section and the results presented. For example, some studies reported to have collected the number of errors (task error rate) or to have used a specific instrument but did not report on these results in the manuscript results section [37], [31,39,41,42].

mHealth solutions have the potential to foster self-assessment and self-management for patients suffering from pain and have a positive impact on their overall functioning and quality of life. The International Association for the Study of Pain has highlighted the mobile apps as a new advantage in the field of pain, highlighting the potential of technology to improve access to health care, contain costs, and improve clinical outcomes, but also calling for the need of studies measuring their efficacy, feasibility, usability, and compliance, and for the involvement of the scientific community reviewing the quality of existing solutions [65].

There are a few limitations to this systematic review. First, the protocol for this systematic review was not registered in a public database. Secondly, the grey literature was not searched. In addition, it lacks the analysis of the impact of the quality of the usability procedures on the results of the usability assessment and the quality of the resulting mobile app. However, the diversity of the procedures used in the manuscripts included in the systematic review makes this analysis difficult.

## 5. Conclusions

This systematic review found 31 manuscripts assessing the usability of a total of 32 pain-related apps, 25 of which were for pain assessment and seven for pain management. A detailed methodological analysis of these manuscripts revealed that several important methodological aspects regarding the assessment of usability for pain-related applications are not being considered. Future developments should be planned and implemented in line with existing guidelines.

## Figures and Tables

**Table 2:** Methodological quality assessment of studies included in the systematic review.

	Methodological Quality Items															Score	%
**Authors**	1	2	3	4	5	6	7	8	9	10	11	12	13	14	15	---	----
Bedson 2019 [44]	0	0	1	1	1	0	0	1	1	0	0	1	0	1	1	8	53.33%
Suso-Ribera 2018 [41]	1	1	0	1	1	0	0	1	1	0	0	1	1	1	1	10	66.67%
Dantas 2016 [29]	1	1	1	1	1	0	1	0	0	0	1	0	0	1	1	9	60.00%
de la Vega 2014 [39]	0	0	1	1	0	1	0	1	1	0	0	0	1	1	0	7	46.67%
de la Vega 2018 [40]	0	0	1	1	0	1	0	0	1	1	0	0	1	1	0	7	46.67%
Diana 2012 [37]	0	0	1	1	1	0	0	0	1	0	0	N/A	1	1	N/A	5	38.46%
Docking 2018 [59]	0	0	1	1	1	0	1	1	1	0	0	0	1	0	0	7	46.67%
Fledderus 2015 [53]	0	0	1	1	1	0	1	1	1	0	0	N/A	1	0	N/A	7	53.85%
Fortier 2016 [51]	0	0	1	0	0	0	1	1	1	0	0	1	0	0	0	5	33.33%
Birnie 2018 [54]	0	0	1	1	1	0	1	0	1	0	0	1	1	1	0	7	46.67%
Boceta 2019 [58]	0	0	0	1	1	0	1	0	1	0	0	1	1	1	1	8	53.33%
Caon 2019 [56]	1	1	0	1	1	0	1	0	0	0	0	N/A	0	1	N/A	6	46.15%
Cardos 2017 [57]	1	1	1	1	1	1	0	0	0	0	0	0	0	0	0	6	40.00%
de Knegt 2016 [34]	0	0	1	1	1	0	1	0	1	0	0	0	1	1	0	7	46.67%
Kaltenhauser 2018 [35]	0	0	0	1	0	0	0	1	0	1	0	0	0	0	0	3	20.00%
Minen 2018 [48]	1	1	1	1	1	0	1	0	1	0	0	N/A	1	1	N/A	9	69.23%
Neubert 2018 [52]	0	0	1	1	1	0	1	1	1	0	0	1	1	1	0	9	60.00%
Reynoldson 2014 [19]	1	1	1	1	1	1	1	0	0	0	0	0	1	1	0	9	60.00%
Spyridonis 2012 [42]	1	1	1	1	1	0	1	0	1	0	0	0	0	1	0	8	53.33%
Spyridonis 2014 [31]	1	1	1	1	1	0	1	1	1	0	0	0	0	1	0	9	60.00%
Stefke 2018 [45]	1	1	1	1	1	0	1	0	0	0	0	1	0	1	1	9	60.00%
Stinson 2013 [49]	0	0	1	1	1	1	1	1	1	0	0	1	1	1	1	11	73.33%
Sun 2018 [55]	1	1	1	1	0	1	1	1	0	0	0	1	1	1	1	11	73.33%
Turner-Bowker 2011 [46]	0	0	1	1	1	0	0	0	1	0	0	0	1	0	0	5	33.33%
Vanderboom 2014 [43]	1	0	1	1	1	1	1	0	1	0	0	1	0	1	1	10	66.67%
Yen 2016 [38]	0	0	1	1	1	0	0	1	1	0	0	0	1	1	0	7	46.67%
Hochstenbach 2016 [50]	0	0	1	1	1	1	1	0	1	0	0	1	0	1	1	9	60.00%
Huguet 2015 [47]	0	0	1	1	1	1	1	0	1	0	0	1	1	1	1	10	66.67%
Jaatun 2013 [36]	0	0	0	1	1	0	0	1	1	0	0	N/A	1	1	N/A	6	46.15%
Jibb 2017 [32]	1	1	1	1	1	0	1	0	1	0	0	1	1	1	N/A	10	71.43%
Jibb 2018 [33]	1	1	1	1	1	0	1	0	1	0	0	1	1	1	1	11	73.33%
Total items scored yes (out of 31)	13	12	26	30	26	9	21	13	24	2	1	14	20	25	11		

**Abbreviated items of the scale:** 1—Valid measurement instruments, 2—reliable measurement instruments, 3—procedures adequate to study’s aims, 4—procedures adequate to the development stage of the product, 5—procedures adequate to the participants’ characteristics, 6—triangulation, 7—analysis adequate to the study’s aims and variables measurement scale, 8—combination of user’s and experts’ evaluation, 9—representativeness of participants (potential users and/or experts) 10—experience of the investigator that conducted the usability evaluation, 11—investigator conducting usability assessment external to the development of the product/service, 12*—assessment in real context or close to real context, 13—number of participants (potential users and/or experts), 14—representativeness of the tasks to perform on the usability evaluation, 15*—continuous and prolonged use of the product. N/A—not-applicable.

## Supplementary Materials

The following are available online at https://www.mdpi.com/1660-4601/17/3/785/s1, Table S1: Overview of the manuscripts included in the systematic review.
