# Supplementary material for: Methodological Quality of Manuscripts Reporting on the Usability of Mobile Applications for Pain Assessment and Management: A Systematic Review
Source: Int J Environ Res Public Health. 2020 Jan 27;17(3):785. doi: 10.3390/ijerph17030785 (PMC7038093; doi:10.3390/ijerph17030785)
Supplement: Supplementary file 1 [file ijerph-17-00785-s001.pdf]

Table S1. Overview of the manuscripts included in the systematic review.

Authors	App	App Aim	Individuals Involved In The Usability Assessment	Use Case	Domain Assessed	Method Of Usability Assessment Used	Usability Outcome	Reviewers Comments
**Dantas_2016 [25]**	Dor de Cabeça; Diário da Dor; Diário da Cefaleia	Assessment	End users<br>N = 22<br><br>Characteristics<br>54.5% (n=12) female and 45.5% (n=10) males; mean (±SD) age of 72.3 (±8.2) years old	Not specified	Efficiency, Effectiveness Satisfaction	International Classification of Functioning - Usability Scale I (ICF-US I);<br><br>Performance evaluation:<br>i) Number of attempts to complete tasks<br>ii) Completion rates<br>iii) Time taken to complete tasks	ICF results:<br>Dor de Cabeça (Mean±sd ICF_US I=25.4 ±0.4),<br>Diário da Dor (Mean±sd ICF_US I=24.7±3.9)<br>Diário da Cefaleia (Mean±sd ICF_US I= 13.6±3.7)<br><br>Time to complete tasks:<br>Diário da Cefaleia (mean±sd=7.1±3.3min)<br>Dor de Cabeça (mean ±sd=6.2 ±2.9 min)<br>Diário da Dor (mean±sd=5.1 ±2.7 min)	
**Reynoldson_2014 [26]**	1. Manage My Pain;<br>2. Pain Scale	Assessment	End users<br>N = 41 (aged 19–59 years, with experience of chronic or recurrent pain)	Record 2 pain episodes (average level of pain they regularly experience and worst pain they have experienced)	Efficiency Effectiveness Satisfaction	1. Time taken to complete a data entry task on the app;<br>2. SUS<br>3. Design questionnaire;<br>4. App use questionnaire	Usability task timings<br>Manage My Pain - ranged from 1′22″ to 6′09″ (mean = 3′01″; SD= 1′14″) for the first entry and from 0′44″ to 4′05″ (mean = 2′00″; SD= 0′49″) for the second entry.<br>Pain Scale app - ranged from 0′02″ to 0′59″ (mean = 0′19″;SD= 0′12″) for the first entry and from 0′02″ to 0′37″ (mean = 0′14″; SD= 0′08″) for the second entry.<br><br>SUS ManageMyPain scores ranged from 57.5 to 100.<br>SUS PainScale scores ranged from 60 to 100.	
**Spyridonis_2014 [28]**	PainDroid	Assessment	End users<br>N = 7 wheelchair users (3 fem; 4 males) with some	1. Start the app	Satisfaction	Questionnaire adapted from SUS	Positive trends in the questions 2 and 3, regarding ease of use.	Total score of the SUS adapted

			arm mobility problems, ranging in age from 21 to 65 years (mean age 41.1)<br><br>Experts<br>N = 2 clinicians (a General Practitioner-GP- and a Rheumatologist-RH)	2. Rotate left/right, rotate up/down<br>3. Re-center<br>4. Zoom in/out and drag<br>5. Reset<br>6. Use above to select pain type and pain location on model<br>7. Save and exit			There was strong disagreement with statements targeting the amount of learning required to have in order to use PainDroid.	questionnaire is not presented
**Jibb_2017 [29]**	Pain Squad+	Intervention	End users<br>N = 16<br>Cycle 1 (n = 4)<br>Cycle 2 (n = 6),<br>Cycle 3 (n = 6)<br><br>Characteristics<br>Adolescents with cancer, ranged in age from 12 to 18 years (mean = 14.8; SD = 2.1), 9 female, 7 male.	Adolescents were asked to complete the app (pain assessment, pain management, and gamification mechanics)	Satisfaction<br>Efficiency<br>Effectiveness	Think aloud approach<br><br>Audio-recording and field notes<br><br>Semi-structured interview<br><br>Recording of time to complete the pain assessment	All participants but one were able to complete the tasks in a single session.<br>Participants across all testing cycles stated that Pain Squad+ was easy to use.<br>The time to complete the pain assessment component of the app across all iterative cycles was 4.3 ± 3.5 minutes (M ± SD; range = 1.9-14.3 minutes).<br>All participants across the 3 cycles were satisfied with the Pain Squad+ app.	
**Jibb_2018 [30]**	Pain Squad+	Intervention	End users<br>N = 20<br><br>Characteristics<br>Adolescents with cancer (12-18 years)	Adolescents were asked to complete the app	Satisfaction<br>Effectiveness	Count number of registers on the app<br><br>Semi-structured telephone interview audio-recorded, with field notes	95% (n=19) of the participants completed the entire pilot study.<br>Every adolescent endorsed the ease of use of Pain Squad+.<br>All participants endorsed pain management advice, design and gamification mechanics.	
**De Knegt_2016 [31]**	STOP-ID!	Assessment	End users<br>N = 40<br><br>Characteristics	1) Patients were asked if they were currently	Efficiency,<br>Effectiveness	Qualitative observations	All participants finished the STOP-ID! The average performance time was 14.6 min (SD¼3.9)	

			Adults with Down syndrome, average age of 43.3 years (SD¼11.7, range: 20–66 years)	experiencing pain;<br>2) locate the pain;<br>3) classify pain intensity;<br>4) assess pain affect selecting Pictograms;<br>5) assess pain Quality selecting Pictograms		Performance evaluation following a form (e.g., presence of distraction or impulsivity);<br>Time to complete the test;<br>Number of times that the instructions needed to be repeated	48% of the participants seemed at times distracted or bored and, 35% appeared to answer at least a few questions impulsively<br><br>7 participants were able to use the tool independently, 4 needed assistance with the device, 16 needed assistance in the questions about the comprehension tests and/or self-report of pain, and 13 needed assistance with both the features.<br><br>Verbal and/or non-verbal requests of assistance were made by 70% of the participants.	
**Kaltenhauser_2018 [32]**	Quiri	Assessment	End users<br>N = 3 adults<br><br>Experts<br>N = 3	Three tasks (not specified)	Effectiveness	Successful completion of tasks<br><br>Discussions with physicians	All participants completed the three tasks.<br><br>The ease of use and short interaction time to complete a measurement were highlighted as particularly valuable by the physicians.	
**Jaatun, E_2013 [33]**	Pain Body Map (PBM)	Assessment	End users<br>N = 10<br><br>Characteristics<br>Patients with advanced cancer (both in the first and second versions of the iPad PBM)	1) Mark the area of pain in the body map,<br>2) Select the intensity of the pain by selecting the image of the corresponding numbered colouring pencil/radio button.	Effectiveness	Observation of task execution – completion rates	First version<br>3 of the 10 patients had no problem using the iPad and complete tasks;<br>5 were able to mark intensity but needed orientation;<br>2 patients were not able to give any input on the iPad (one was too frail and the other was unable to follow instructions).<br><br>Second Version	

							8 participants completed the tasks without support;<br>2 patients were not able to fill in anything at all due to drowsiness /sleepiness and having problems following instructions.	
**Diana_2012 [34]**	No reference to app name	Assessment	End users<br>N = 5 (woman with fibromyalgia)	Not specified	Satisfaction<br>Effectiveness<br>Efficiency*	Mock-ups of the app<br>Questionnaire<br>Recording of the sessions<br>Success/failure rates<br>Time to complete tasks	All the participants were able to give ratings in the three scales.<br><br>100% of the users found the device was easy to use<br><br>75% of the users thought they would be able to use the device.<br><br>75% of the users had difficulties to understand how to advance (did not understand the metaphor of the button, represented by an arrow.	The authors only present the mean time of each session, they make no reference to the time to complete the tasks
**Caon_2019 [35]**	My Pain Coach	Assessment	End users<br>N = 6<br>Characteristics<br>People without any chronic pain condition	1) Register;<br>2) create a new "pain entry" with Tangible Interface;<br>3) create a new "pain entry" with Touch screen;<br>4) View reports and visualize "Pain map";<br>5) Insert a password for doctors.	Satisfaction<br>Efficiency<br>Effectiveness	SUS<br><br>Recording of time to complete tasks<br><br>Completion rates of the tasks	The SUS score assessed usability as "excellent" (mean result of all users was 82,5±5,7); users perceived this system as easy-to-use.<br><br>Time to complete tasks (in seconds) ± SD (mean of the 6 users):<br>task1 – 141±72;<br>task2 – 229±71;<br>task3 - 153±55;<br>task4 - 31±14<br>task5 - 33±13<br><br>Success rate - 100% (all participants completed all tasks)	

**Birnie, K_2018 [36]**	Achy Penguin	Intervention	End users<br>N = 20 (Cycle 1, n=6, Cycle 2 and 3, n= 7)<br>Characteristics<br>4 to 7 year-old children (mean age 5.8 years)<br><br>Experts<br>N = 2<br>Characteristics<br>Child life specialists at Seattle Children's Hospital reviewed and tested the initial app prototype	The participants progressed through all aspects of the app, including pain assessment (pain location and intensity) and pain management strategies	Satisfaction<br>Efficiency<br>Effectiveness	Qualitative interview;<br>Think aloud approach (likes, dislikes, and difficulties using the app);<br>Field notes on children's responses (taken by research assistants);<br>Recording of the length of time children engaged with app features.	Time engagement with app features:<br>Pain intensity (M, SD, range in seconds):<br>Cycle1 - 44.2, ±22.9, 30–90<br>Cycle2 - 21.7, ±20.1, 10–60<br>Cycle3 - 24.7, ±18.2, 10–60<br><br>Pain location (M, SD, range in seconds):<br>Cycle1 - 90.0, ±53.7, 30–180<br>Cycle2 - 23.6, ±18.4, 15–60<br>Cycle3 - 40.0, ±30.3, 10–90<br><br>Self-management strategies (M, SD, range in minutes):<br>Cycle1 – 7.7, ±2.7, 3–10<br>Cycle2 - 9.1, ±3.5, 6–16<br>Cycle3 - 14.0, ±1.8, 11–16	
**Sun_2018 [37]**	Panda	Assessment	End users<br>N = 17<br>• 12 parents (4 in round 1, 3 in round 2, 5 in round 3),<br>• 5 adolescents (round 3)<br><br>Experts<br>N = 13 nurses (6 in round 1, 7 in round 2)	a) Enter child and scheduled medication<br>b) Respond to app alert, performing a pain assessment and complete checks for medication administration, and then edit the status of the medication<br>c) Respond to a notification when a medication has already been given,	Efficiency<br>Effectiveness<br>Satisfaction	1) Task errors rate<br>2) Task completion rate<br>3) Time to complete tasks<br>4) "think aloud" and was audio-recording of the sessions.<br>5) Computer System Usability Questionnaire (CSUQ)<br>5) Unstructured interview	All users were able to successfully complete tasks a) and b).<br><br>Nurses tended to perform better in terms of task completion rate and time, as well as task error rates<br><br>Users' CSUQ responses had median [IQR] score of 2 [1-4]; 67% of users indicated that they would use Panda for management of postoperative pain.<br><br>93% of participants reported the app was easy to use	

				d) Schedule medication, edit and delete existing medication.				
**Yen_2016 [38]**	RhEumAtic Disease activitY (READY)	Assessment	End users<br>N = 33 patients<br>N = 15 physicians	1) Completing questionnaires, 2) Navigate the system, 3) Patient and physician assessment of pain 4) Documenting medications, 5) interpret results	Satisfaction<br>Effectiveness<br>Efficiency	1) Think Aloud protocol<br>2) Video-recording of interactions with READY<br>3) Comparison of the time using READY and paper evaluation.	Usability issues were found in touchscreen sensitivity, interface design, interactive features, and instruction and error messages. Despite these issues, 75.76% patients reported they liked READY.<br>About 37% of patients found touch screen sensitivity was not sensitive enough to complete an action. Physicians faced similar issues.<br><br>Comparing the times, results show that patients spent more time on READY than paper (mean=4.39 mins, SD=2.29 vs. mean=2.26 mins, SD=1.36, p=0.002). In this matter, physicians also revealed concerns about clinical workflow.	
**De La Veja_2014 [39]**	Painometer	Assessment	End users<br>N = 14 (non-professionals, with history of pain)<br><br>Experts<br>N = 19 (professionals)	Assessing pain intensity with the scales; see list of scales; see description of the scale and instructions; choose or change scale.	Satisfaction<br>Effectiveness	Semi-structured interview<br><br>Open-ended questions<br><br>Recording of errors<br><br>Recording of sessions and field notes	Most of health care professionals (n=18, 95%) preferred Painometer to traditional versions of the scales.<br><br>Participants completed the tasks, but 3 (1 non-professional and 2 professionals) had some difficulties with the buttons size.	Results of the task errors are not presented

						Tasks completion rates<br>Think Aloud approach		
**De La Veja_2018 [40]**	Fibroline	Intervention	End users<br>N = 25<br>Characteristics<br>13 to 24 years (mean age = 18.24; SD = 4.02), 8 males (32%) and 17 females (68%) with fibromyalgia	Log in the app, change settings, access certain resources—read a PDF file, listen to an audio, and watch a video, set an alarm for medication, go back to the main screen	Satisfaction	Semi structured interview (Yes/No questions and open-ended questions)<br>Field notes of the sessions and recording of errors<br>Think Aloud approach	All participants (100%) rated Fibroline as "Easy to use."<br><br>All participants (100%) would use Fibroline if they needed it.<br><br>Problems reported using the app - finding button to access chart, setting alarm, choose to see some content in text or video format.	The results of the recorded mistakes are not presented.
**Suso-Ribera_2018 [41]**	Pain Monitor	Assessment	End Users<br>N = 38 (patients with chronic pain; mean age 42.74, SD = 9.92)<br><br>Experts<br>N = 7 (two psychologists and five physicians)	Evaluate pain (twice a day for 30 days)	Satisfaction	System Usability Scale (SUS)	All participants (100%) found the app extremely easy to use.	The total score of SUS is not presented
**Spyridonis_2012 [42]**	PainDroid	Assessment	End users<br>N = 7 (wheelchair users over 18 years old)	1. Start the app<br>2. Rotate left/right, rotate up/down<br>3. Re-center<br>4. Zoom in/out and drag 5. Reset<br>6. Use above to select pain type and pain location on model<br>7. Save and exit	Satisfaction	Questionnaire adapted from SUS	The wheelchair users' evaluation highlighted positive bias in respect of the application's usability and functionality<br><br>There was a positive trend in the question 3, referring to ease of use (Mean=4 St. Dev.=1)	Total score of the SUS adapted questionnaire is not presented

**Vanderboom_2014 [43]**	My Pain Diary	Assessment	End users<br>N = 20 (patients who had complete an existing fibromyalgia treatment)	Enter the app to rate pain, fatigue and activity.	Satisfaction	1) Perceived Ease of Use (PEU) scale<br><br>2) Perceived Usefulness (PU) scale<br><br>3) Frequency of use	80% of the participants found the device easy to use, 75% found it easy to interact with and 70% thought it was easy to configure<br><br>75% of the users felt the app was useful to manage their condition<br><br>Frequency of use over 7 days (in days) – 5.2±2.2(M±SD)	
**Bedson, J_2019 [44]**	Keele Pain Recorder (KPR)	Assessment	End Users<br>N= 21 (13 females and 8 males); median age 62 (IQR 50 to 70)<br><br>Experts<br>N = 17 (General Practitioners, physiotherapists, research nurses, primary care researchers, research facilitators, and an IT manager)	Enter pain recordings (twice daily for a period of 4 weeks)	Effectiveness<br>Satisfaction	Completion rates<br><br>Follow-up questionnaire<br><br>Think aloud approach (Workshop)<br><br>Semi-structured telephone interviews	Participants entered 862 records. Frequency of app use - 9/18 daily/often, 0/18 never.<br>Patients found the KPR easy to use.<br>General Practitioners found the graphical output easy to interpret.	
**Stefke_2018 [45]**	Migraine Monitor	Assessment	End users<br>N = 21 subjects (71% female, age 31±13 (M±SD))	Use the migraine diary function over a period of two weeks to register migraine attacks and to document daily mood changes.	Satisfaction	SUS	Overall, the application reached a SUS score of 91.67±6.96 (M ± SD).<br><br>The average responses to the question "I think that I would use this system frequently" were considerably worse (2.71±1.41) compared to the average score of all other questions (3.77±0.52).	
**Turner-Bowker_2011 [46]**	HEADACHE-CAT	Assessment	End users<br>N = 9 headache sufferers (6 women and 3 men, age range, 27-54; mean, 38 years.	Participants navigated the survey and responded to the	Satisfaction	1) Structured interviews to gather user feedback on interface, visual	Participants reported that the stylus was ''easy'' or ''extremely easy'' to use.	

				survey questions.		design and navigation;<br>2) Observation of the interviews and the interaction of users with the app;<br>3) Video recording of the process.	Participants were easily able to determine how to begin the survey again, close the application, return to a previous question, and proceed to a subsequent question.	
**Huguet_2015 [47]**	myWHI diary (my Wireless Headache Intervention diary)	Assessment	End users<br>Cycle 1 (N=11)<br>Cycle 2 (N=19)<br>Cycle 3 (N=13)<br><br>Characteristics<br>Adolescents and young adults, age range 14-28years old.	Use the diary for 14 days.<br>Report occurrence of headache: start and ending time, intensity, location, trigger(s), headache quality, highest pain intensity.<br>Enter additional information (e.g., overall mood, hours and quality of sleep).	Effectiveness<br>Satisfaction	Closed-ended questionnaire<br><br>Adherence and completion rates<br><br>Semi-structured interviews in the end of app use	A minority of participants completed all daily entries (18%, n=2) in cycle 1.<br>In cycle 2, participants completed headache entries closer to the pain beginning time than in Cycle 1 (Cycle 2 mdn=3.83h vs Cycle 1 mdn=13.59h). Adherence was also improved, 26% of participants (n=5) completed all 14 daily entries.<br>Cycle 3, the majority of participants' headache entries during Cycle 3 were made on the same day that the episode occurred.<br>In all three cycles, most participants rated the app as easy to use and navigate.	
**Minen_2018 [48]**	RELAXaHEAD	Intervention	End users<br>N = 12<br><br>Characteristics<br>Adults with headache, 80% female (mean age was 37.5615.97, range 20–74 years).	Entry of daily headache diary data;<br>Enter medications used;<br>Register sleep data	Satisfaction	Think Aloud approach and Scripted protocol<br><br>Recording of the sessions<br><br>Likert-scaled questions about satisfaction	75% of the participants thought the diary was easy to use,<br>75% agreed that it was relevant to help track their headaches,<br>83% thought the information in the app was easy to understand,<br>75% agreed that the app kept their interest and attention during the session.	

							92% of the participants indicated that they would use the app again.	
**Stinson_2013 [49]**	Pain Squad	Assessment	End users<br>Low-fidelity<br>N = 15 adolescents (5 per cycle)<br>High-fidelity<br>N = 18 (1st cycle)<br>N = 8 (2nd cycle)<br><br>Experts<br>N = 20<br>Characteristics<br>10 pediatric oncologists<br>10 pediatric pain experts	To complete the app, recording their own pain.	Satisfaction<br>Effectiveness	Semi-structured, audio-taped interviews<br><br>Register of entries in the system	Low-Fidelity<br>Participants made positive comments on the appearance of the app screenshots;<br>Participants suggested several changes to improve usability of the game-based app<br>High Fidelity<br>Every adolescent interviewed said they would use the app daily for an extended period;<br>All participants found the app to be “easy to understand” and “easy to navigate”.<br>100% entries were completed within the 2-week period.	
**Hochstenbach_2016 [50]**	No reference to app name	Intervention	End users<br>N = 11 (patients with moderate to severe cancer pain, 5 males, 6 females)	1) Register pain, adverse effects, interference of pain with activity or sleep, and satisfaction with pain treatment; 2) register pain medication taken.	Satisfaction<br>Effectiveness	Questionnaire to assess learnability, usability and desirability (Likert-scale)<br><br>Completion rates<br><br>Semi-structured interview	Patient quickly learned how to use the app (mean=4.8, SD=0.4).<br><br>Average completion rates were 76.8% for pain monitoring, 50.4% for medication monitoring and 100% for education sessions.<br><br>Patients completed, on average, 43 (SD 12.4) out of the 56 diaries.<br><br>Overall, patient considered the app clear and simple.	
**Fortier_2016 [51]**	Pain Buddy	Intervention	End users<br>N = 12<br>Characteristics<br>Children with diagnose of cancer, ages 8-18 (12.33+/-3.42; 58% male)	Complete the symptom diary twice daily for a 10 day period	Satisfaction<br>Effectiveness	Queries with a likert-type scale about usefulness and ease of use;	Mean number of completed diaries – 19.58 (SD=4.64; range 13-27)<br><br>Participants were satisfied with the app	

						Open-ended questions<br>Completion rates of the diaries		
**Neubert_2018 [52]**	No reference to app name	Assessment	End users<br>Study 1<br>N = 52 patients with chronic pain<br><br>Study 2<br>N = 51 patients with chronic pain and<br>N = 4 doctors	1) Make a correct symptom drawing on each view of the body outline showing the respective body region,<br>2) Specify the symptom choosing descriptors from a list,<br>3) Specify any pain-related symptom,<br>4) Rate the intensity of pain.	Satisfaction	**End users:** usability questionnaire, with 8 Likert-type questions and 2 open questions.<br><br>**Experts:** Web-based survey comprising the questionnaires:<br>1) SUS<br>2) Attrakdiff 2<br>3) The ISONORM 9241/10 questionnaire.	End users<br>In study 1, 87% (45/52) of the study patients were content with the body outline<br><br>Experts<br>Score of 75.63 on the SUS indicating an overall good usability	
**Fledderus_2015 [54]**	No reference to app name	Intervention	End users<br>N = 1<br>Characteristics<br>Patient with chronic pain<br><br>Experts<br>N = 5<br>Characteristics<br>1 target group expert and 4 eHealth experts (mean age - 38, SD 12)	Scenarios with tasks/problems to solve<br><br>(Tasks are not specified)	Satisfaction	Think Aloud approach (verbalizing thoughts during the scenarios)<br><br>Audio recording of the sessions<br><br>Evaluation of comments as positive, negative or neutral.	Ease of use of the app (e.g., clear navigation, clear buttons, simple) and the design (e.g., fresh, calm) were rated positively.<br><br>Usefulness of the features also had positive comments.<br><br>Most negative comments were about the quality of the system as some (technical errors and unclear icons).	
**Cardos_2017 [55]**	BIO-H App	Intervention	End users<br>N = 16	1) opening the intervention app;	Effectiveness<br>Satisfaction	System Usability Scale	A mean score of 93.44 (SD=7.06) out of 100 was found for the	

			Characteristics<br>Females (mean age = 42,43)	2) read the information about the intervention;<br>3) fill pre-intervention survey;<br>4) listen to the audio relaxation exercise;<br>5) fill out the post-intervention survey		Structured Questionnaire<br><br>Task completion rates	overall usability based on the participants' SUS ratings.<br><br>People found the intervention component to be useful (m=8.63 out of 10; SD = 1.14) and they indicated a high level of willingness to access the app in the future (m=8.56; out of 10; SD=1.71).<br><br>All tasks were completed successfully, and participants commented on how easy the interface was to navigate.	
**Boceta, J_2019 [56]**	App INES·DIO	Assessment	End users<br>N = 175 physicians	Open a new profile for each patient and go through four sequential steps to collect relevant clinical information for an enhanced diagnosis procedure of BTcP.	Satisfaction<br>Effectiveness	Structured questionnaire<br><br>Assessment test related to usability (included as last feature of the app)<br><br>Follow-up questions about the app design, overall usefulness, and use intention<br><br>Utilization rates of the app and each assessment scale	Most clinicians (157/175, 89.7%) concluded that the mobile app is well designed and easy to use<br><br>94.9% (166/175) of participants would likely/most likely recommend the use of App INES·DIO.<br><br>The app was used as often as two or three times a week by 41.7% (74/175) of clinicians.<br><br>Pain-rating and functional assessment scales were the most frequent tools used by clinicians (93.3%, 163/175)	Boceta, J_2019 [56]
**Docking_2018 [57]**	iPhone pain app	Assessment	End users<br>N = 24<br>Characteristics<br>Trainee paramedics (46% females and 54% males)<br><br>Experts	Participants were asked to work their way through the app, trialling out different routes.	Satisfaction	4 focus groups with trainee paramedics<br><br>Direct questions related to changes/ recommendations	Focus group revelled concerns around the usability of technological devices within the paramedic setting and whether it is professional to use them in practice.	

			N= 7 Characteristics Paramedics (86% males and 14% females)			Delphi Panel of Paramedic Experts	Some suggestions were related to making the app more intuitive, in particular the location of the “next” and “back” buttons and the correction of some links.	
